# The Kidney Protective Effects of the Sodium–Glucose Cotransporter-2 Inhibitor, Dapagliflozin, Are Present in Patients With CKD Treated With Mineralocorticoid Receptor Antagonists

**DOI:** 10.1016/j.ekir.2021.12.013

**Published:** 2021-12-14

**Authors:** Michele Provenzano, Niels Jongs, Priya Vart, Bergur V. Stefánsson, Glenn M. Chertow, Anna Maria Langkilde, John J.V. McMurray, Ricardo Correa-Rotter, Peter Rossing, C. David Sjöström, Robert D. Toto, David C. Wheeler, Hiddo J.L. Heerspink, DAPA-CKD Trial Committees and Investigators

**Affiliations:** 1 Nephrology Unit, Department of Advanced Medical and Surgical Sciences, University of Campania “Luigi Vanvitelli,” Naples, Italy; 2 Department of Clinical Pharmacy and Pharmacology, University Medical Centre Groningen, University of Groningen, Groningen, The Netherlands; 3 Late-Stage Development, Cardiovascular, Renal and Metabolism, BioPharmaceuticals R&D, AstraZeneca, Gothenburg, Sweden; 4 Departments of Medicine and Epidemiology and Population Health, Stanford University School of Medicine, Stanford, California, USA; 5 Institute of Cardiovascular and Medical Sciences, University of Glasgow, Glasgow, UK; 6 Instituto Nacional de Ciencias Médicas y Nutrición Salvador Zubirán, Mexico City, Mexico; 7 Steno Diabetes Center Copenhagen, Gentofte, Denmark; 8 Department of Clinical Medicine, University of Copenhagen, Copenhagen, Denmark; 9 Department of Internal Medicine, UT Southwestern Medical Center, Dallas, Texas, USA; 10 Department of Renal Medicine, University College London, London, UK; 11 The George Institute for Global Health, Sydney, Australia

**Keywords:** chronic kidney disease, DAPA-CKD, dapagliflozin, hyperkalemia, mineralocorticoid receptor antagonists, sodium–glucose cotransporter-2 inhibitor

## Abstract

**Introduction:**

Mineralocorticoid receptor antagonists (MRAs) and sodium–glucose cotransporter-2 (SGLT2) inhibitors reduce the risk of kidney failure in chronic kidney disease (CKD). We performed an analysis of the Dapagliflozin and Prevention of Adverse Outcomes in Chronic Kidney Disease (DAPA-CKD) trial by baseline conventional MRA (spironolactone and eplerenone) prescription.

**Methods:**

Participants with CKD (estimated glomerular filtration rate [eGFR] 25–75 ml/min per 1.73 m^2^; urinary albumin-to-creatinine ratio 200–500 mg/g), with or without type 2 diabetes, were randomized 1:1 to dapagliflozin 10 mg or placebo, once daily. The primary outcome was a composite of sustained ≥50% eGFR decline, end-stage kidney disease, or kidney or cardiovascular (CV) death. A prespecified kidney-specific secondary outcome was as the primary outcome but without CV death. Hyperkalemia (serum potassium ≥6.0 mmol/l) was an exploratory end point. Time-to-event analyses (proportional hazards [Cox] regression) assessed dapagliflozin versus placebo in patient subgroups defined by baseline conventional MRA use.

**Results:**

A total of 229 of 4304 DAPA-CKD participants (5.3%) were receiving conventional MRAs at baseline (dapagliflozin *n =* 109, placebo *n =* 120). The effect of dapagliflozin on the primary outcome was consistent in participants prescribed (hazard ratio [HR] 0.76, 95% CI 0.40–1.47) and not prescribed (HR 0.60, 95% CI 0.50–0.72, *P*-interaction = 0.59) MRAs. This consistency was maintained for the kidney-specific outcome. The effect of dapagliflozin on hyperkalemia (HR 0.87, 95% CI 0.70–1.09) was consistent among those prescribed (HR 0.94, 95% CI 0.41–2.20) and not prescribed (HR 0.87, 95% CI 0.69–1.10, *P*-interaction = 0.96) MRAs. Adverse events (AEs) leading to discontinuation and serious AEs were similar between treatment groups, regardless of baseline MRA prescription.

**Conclusion:**

Dapagliflozin was similarly safe and efficacious in reducing major adverse kidney outcomes in participants with CKD who were or were not prescribed MRAs at baseline.

See Commentary on Page 371

Angiotensin-converting enzyme inhibitors and angiotensin receptor blockers have been the mainstay of treatment for the prevention of kidney failure in patients with CKD over the last 2 decades. However, despite use of these agents, the risk of kidney failure remains high. New therapeutic options to slow progressive loss of kidney function in patients with CKD with and without diabetes have emerged.^1^

MRAs reduce albuminuria in patients with CKD, but clinical trials to establish their effects on major kidney outcomes were lacking until recently.^2^ The Finerenone in Reducing Kidney Failure and Disease Progression in Diabetic Kidney Disease (FIDELIO-DKD) and Finerenone in Reducing CV Mortality and Morbidity in Diabetic Kidney Disease (FIGARO-DKD) trials demonstrated that the nonsteroidal MRA, finerenone, significantly reduces the risk of a composite kidney outcome of 40% eGFR decline, end-stage kidney disease, or renal death by 18% and 13%, respectively, in patients with type 2 diabetes and CKD.^3^^,^^4^ In addition, SGLT2 inhibitors reduce the risk of kidney failure and slow the progression of eGFR decline.^5^^,^^6^ These benefits were initially demonstrated in the Canagliflozin and Renal Events in Diabetes with Established Nephropathy Clinical Evaluation (CREDENCE) trial, which recruited patients with type 2 diabetes and CKD. The DAPA-CKD trial confirmed and extended these findings by demonstrating that the 39% reduction in risk of the composite kidney end point was similar in patients with CKD with or without type 2 diabetes.^7^

The DAPA-CKD trial enrolled patients treated with angiotensin-converting enzyme inhibitors and angiotensin receptor blockers and also allowed treatment with conventional MRAs (spironolactone and eplerenone). We report the efficacy and safety of dapagliflozin in patients with CKD who were or were not treated with MRAs in DAPA-CKD.

## Methods

DAPA-CKD was a randomized, double-blind, placebo-controlled, multicenter clinical trial. The design and main results have been published previously.^6^^,^^8^ In brief, adults with or without type 2 diabetes, eGFR 25 to 75 ml/min per 1.73 m^2^, and urine albumin-to-creatinine ratio (UACR) 200 to 5000 mg/g were eligible for participation. All participants used a stable dose of an angiotensin-converting enzyme inhibitor or angiotensin receptor blocker for ≥4 weeks unless medically contraindicated. Use of conventional steroidal MRAs (spironolactone and eplerenone) was permitted. A complete list of inclusion and exclusion criteria and the trial protocol have been previously published.^8^ Participants were randomly assigned to dapagliflozin 10 mg once daily or matching placebo. After randomization, in-person study visits were performed after 2 weeks and 2, 4, and 8 months and at 4-month intervals thereafter.

The primary composite end point was time to 50% decline in eGFR (confirmed by a second measurement after at least 28 days), onset of kidney failure (defined as maintenance dialysis for at least 28 days, kidney transplantation, or eGFR <15 ml/min per 1.73 m^2^ confirmed by a second measurement after at least 28 days), or death from a kidney or CV cause. A secondary, kidney-specific end point was defined in the same way as the primary end point but excluding CV death. The 2 other secondary end points were heart failure hospitalization or CV death, and all-cause mortality. Change in eGFR slope was prespecified as an exploratory efficacy end point. All efficacy end points were adjudicated by a masked, independent event adjudication committee, except for the quantitative assessments of eGFR-based end points—50% eGFR decline, eGFR <15 ml/min per 1.73 m^2^, or eGFR slope—which were obtained from our central laboratory.

We conducted time-to-event analyses to assess effects of dapagliflozin versus placebo using a proportional hazards (Cox) regression stratified by randomization factors (diabetes status and UACR), adjusting for baseline eGFR. We analyzed the effects of dapagliflozin on the mean on-treatment eGFR slope by fitting a two-slope mixed effects linear spline model (with a knot at week 2) with a random intercept and random slopes for treatment. Details of the analytical approach have been published previously.^6^^,^^8^

## Results

Out of 4304 participants, 229 (5.3%) were treated with conventional MRAs at baseline; 109 were assigned to dapagliflozin and 120 to placebo (Table 1). Compared with patients not treated with MRAs at baseline, those treated with MRAs were more frequently male and more likely to have a history of heart failure and type 2 diabetes, along with a higher body mass index and higher UACR. Participants treated with MRAs were also more likely to be treated with other CV therapies including diuretics, beta-blockers, statins, and antithrombotic medications (Table 1).

**Table 1:** Baseline characteristics by baseline MRA use

Characteristic	Dapagliflozin		Placebo	
	MRA use (*n =* 109)	No MRA use (*n =* 2043)	MRA use (*n =* 120)	No MRA use (*n =* 2032)
Age, yr	61.8 ± 10.6	61.8 ± 12.1	62.6 ± 10.7	61.8 ± 12.2
Female sex*, n* (%)	30 (27.5)	679 (33.2)	32 (26.7)	684 (33.7)
BMI, kg/m^2^	32.2 ± 7.9	29.2 ± 5.9	33.1 ± 7.3	29.4 ± 6.2
Blood pressure, mm Hg				
Systolic	138.1 ± 19.2	136.7 ± 17.4	137.9 ± 19.7	137.4 ± 17.2
Diastolic	76.5 ± 12.1	77.5 ± 10.6	77.6 ± 10.8	77.5 ± 10.3
eGFR, ml/min per 1.73 m^2^	44.3 ± 12.4	43.2 ± 12.3	43.1 ± 11.4	43.0 ± 12.5
Median UACR, mg/g [IQR]	1165 [541–1977]	951 [469–1894]	1005 [477–2010]	931 [484–1861]
HbA1c, %	7.1 ± 1.5	7.1 ± 1.7	7.4 ± 1.9	7.0 ± 1.7
Type 2 diabetes, *n* (%)	82 (75.2)	1373 (67.2)	89 (74.2)	1362 (67.0)
Heart failure history, *n* (%)	36 (33.0)	199 (9.7)	45 (37.5)	188 (9.3)
Baseline medication use, *n* (%)				
MRA^a^				
Spironolactone	90 (82.6)	0 (0)	106 (88.3)	0 (0)
Eplerenone	18 (16.5)	0 (0)	14 (11.7)	0 (0)
Loop diuretic	62 (56.9)	461 (22.6)	67 (55.8)	466 (22.9)
Thiazide diuretic	36 (33.0)	416 (20.4)	28 (23.3)	426 (21.0)
β-blocker	73 (67.0)	773 (37.8)	82 (68.3)	752 (37.0)
Statin	87 (79.8)	1308 (64.0)	93 (77.5)	1306 (64.3)
Antithrombotic agent	70 (64.2)	952 (46.6)	87 (72.5)	933 (45.9)

BMI, body mass index; eGFR, estimated glomerular filtration rate; HbA1c, glycated hemoglobin; IQR, interquartile range; MRA, mineralocorticoid receptor antagonist; UACR, urinary albumin-to-creatinine ratio.

Data are presented as mean ± SD unless otherwise stated.

a One patient in the dapagliflozin group was treated with an MRA, but the type of MRA (spironolactone or eplerenone) was unknown.

**Figure 1:**
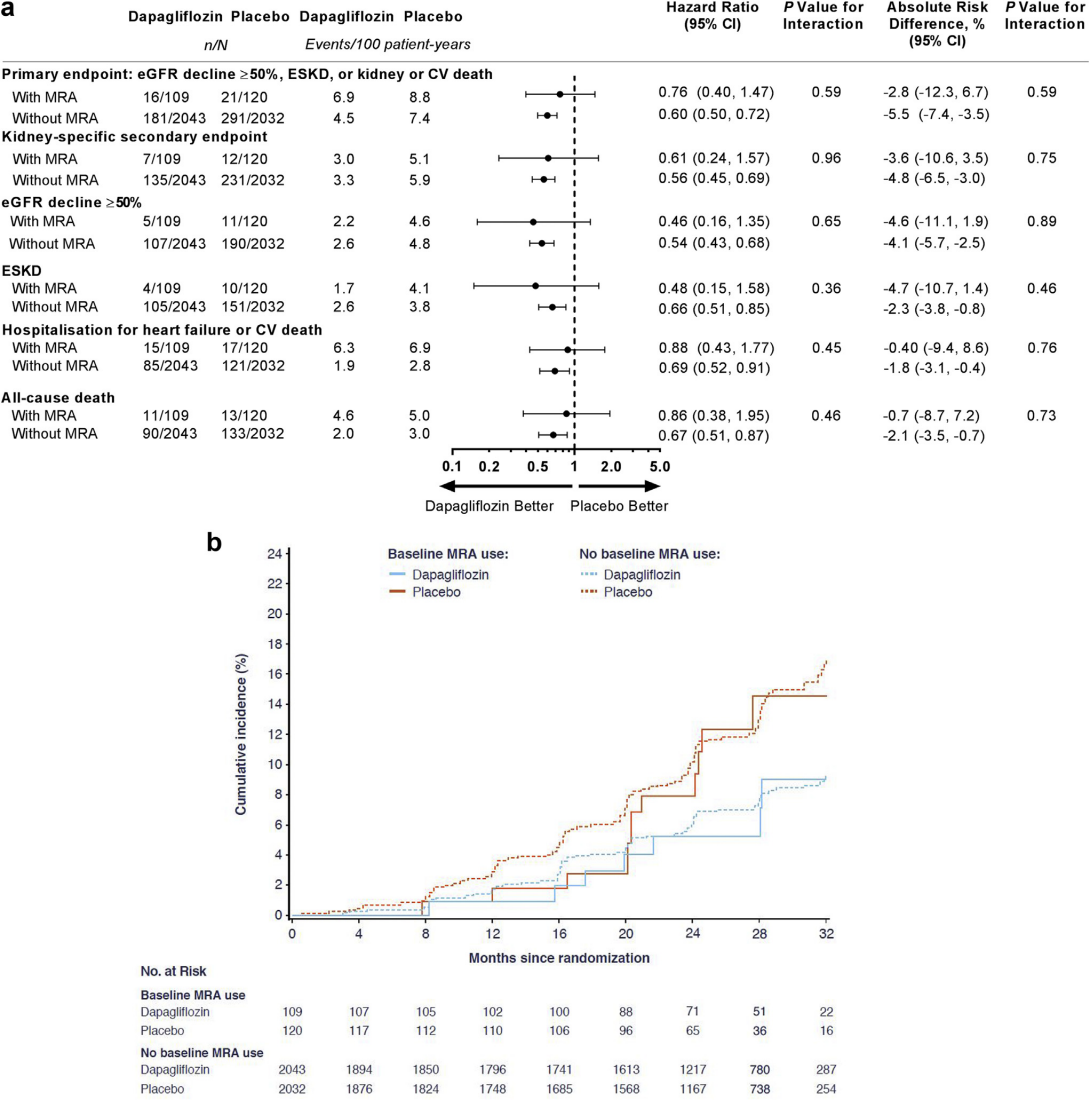
Outcomes for participants with and without MRA use at baseline. (a) Outcomes for the primary composite, the secondary kidney-specific composite outcome, eGFR decline ≥50%, ESKD, hospitalization for heart failure or CV death, and all-cause death. (b) Cumulative incidence of the secondary kidney-specific outcome. CV, cardiovascular; eGFR, estimated glomerular filtration rate; ESKD, end-stage kidney disease; MRA, mineralocorticoid receptor antagonist.

The relative risk reduction for the primary composite end point achieved with dapagliflozin versus placebo was similar in participants treated with MRAs at baseline (HR 0.76, 95% CI 0.40–1.47) compared with those not treated (HR 0.60, 95% CI 0.50–0.72*, P*-interaction = 0.59) (Figure 1a). Similarly, the benefit of dapagliflozin versus placebo on the secondary composite kidney end point did not differ in patients treated with MRAs (HR 0.61, 95% CI 0.24–1.57) and those who were not (HR 0.56, 95% CI 0.45–0.69, *P*-interaction = 0.96) (Figure 1a and b). Benefits of dapagliflozin on the individual components of the primary composite end point were also consistent by baseline MRA use (Figure 1a). The benefits of dapagliflozin on the other 2 secondary end points (heart failure hospitalization or CV death and all-cause mortality) were also consistent among those treated or not treated with MRAs at baseline (Figure 1a).

**Figure 2:**
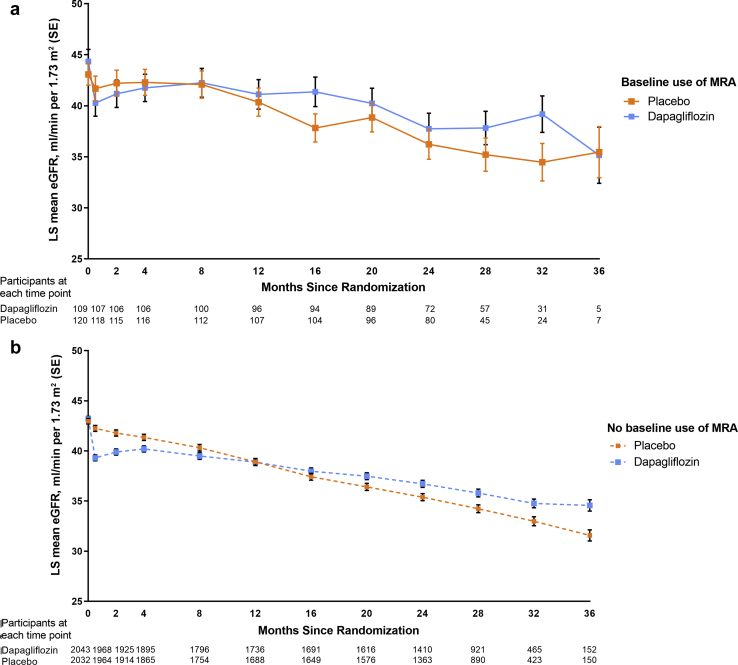
eGFR over time in participants with and without MRA use at baseline. (a) With MRA use at baseline. (b) Without MRA use at baseline. eGFR, estimated glomerular filtration rate; LS, least-squared; MRA, mineralocorticoid receptor antagonist.

**Figure 3:**
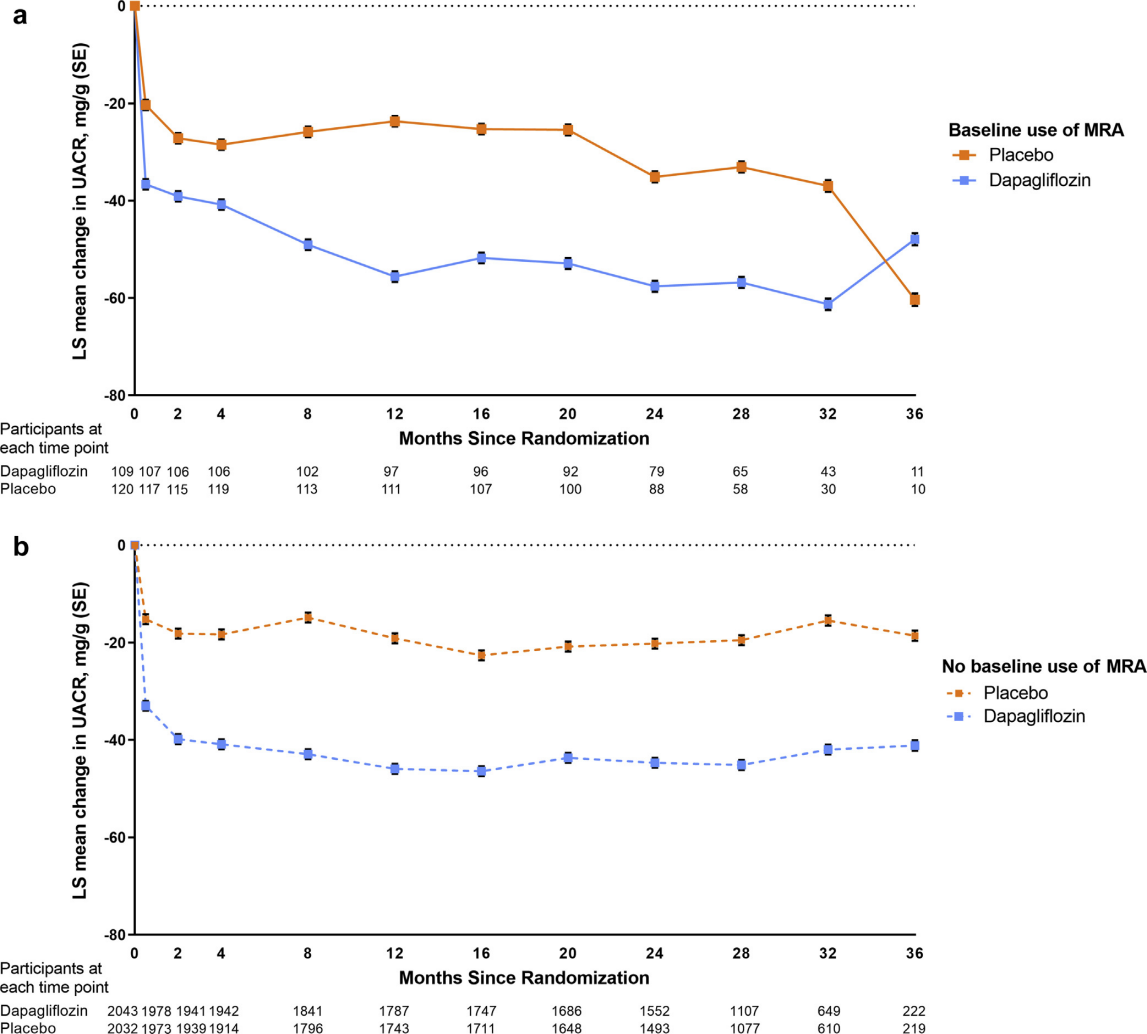
UACR over time in participants with and without MRA use at baseline. (a) With MRA use at baseline. (b) Without MRA use at baseline. MRA, mineralocorticoid receptor antagonists; LS, least-squared; UACR, urine albumin-to creatinine ratio.

Compared with placebo, dapagliflozin caused an acute decline in eGFR from baseline to week 2, and this effect was similar irrespective of MRA use. Thereafter, dapagliflozin reduced the rate of eGFR decline to a similar extent in patients treated with MRAs at baseline (difference [placebo vs. dapagliflozin] 2.0 ml/min per 1.73 m^2^/yr, 95% CI 0.7–3.3) (Figure 2a) and those not treated (difference [placebo vs dapagliflozin] 1.9 ml/min per 1.73 m^2^/yr, 95% CI 1.6–2.2, *P*-interaction = 0.48) (Figure 2b). Effects of dapagliflozin on UACR were also consistent according to baseline MRA use (Figure 3a and 3b).

With respect to safety, serious AEs were more frequently reported in participants treated with MRAs compared with those not treated with MRAs at baseline, but this was true for both the dapagliflozin and placebo groups. The incidence of AEs leading to study drug discontinuation or serious AEs did not differ between dapagliflozin and placebo groups and was similar in participants treated and not treated with MRAs at baseline (Table 2). The effect of dapagliflozin versus placebo on hyperkalemia (HR 0.87, 95% CI 0.70–1.09) was consistent among those treated with MRAs at baseline (HR 0.94, 95% CI 0.41–2.20) compared with those not treated (HR 0.87, 95% CI 0.69–1.10, *P-*interaction = 0.96).

**Table 2:** Safety outcomes by baseline MRA use

Safety outcomes, *n* (%)	Baseline MRA use		No baseline MRA use	
	Dapagliflozin (*n =* 109)	Placebo (*n =* 120)	Dapagliflozin (*n =* 2040)	Placebo (*n =* 2029)
Study drug discontinuation because of adverse event	4 (3.7)	6 (5.0)	114 (5.6)	117 (5.8)
Any serious adverse event^a^	43 (39.4)	51 (42.5)	551 (27.0)	623 (30.7)

MRA, mineralocorticoid receptor antagonist.

a Includes death; safety outcomes were analyzed in the safety population (*N* = 4298).

## Discussion

We demonstrated that the benefits of dapagliflozin in patients with CKD with and without type 2 diabetes on the primary composite and other kidney end points were consistent in patients who were or were not treated with conventional steroidal MRAs at baseline. The safety and tolerability of dapagliflozin was also consistent irrespective of MRA use. These data suggest that the kidney protective effects of both drug classes are possibly complementary and support future trials to prospectively assess the efficacy and safety of combination therapy with SGLT2 inhibitors and MRAs.

The nephroprotective effects of SGLT2 inhibitors and MRAs can be explained through common and distinct mechanistic pathways. Both classes of agents exert hemodynamic effects and result in a reduction in intraglomerular pressure, which likely contributes to long-term stabilization of kidney function.^9^ The blood pressure lowering effects of both drug classes may exert additional benefit. There are also possibly complementary mechanisms. SGLT2 inhibitors improve tubular oxygenation and reduce tubular hypoxic stress.^10^ MRAs reduce renal fibrosis and glomerulosclerosis by deactivating the mineralocorticoid receptor in fibroblasts.^11^^,^^12^ Experimental and clinical studies with the MRA, finerenone, support potential complimentary effects. A *post hoc* analysis from the FIDELIO-DKD trial demonstrated that finerenone compared with placebo reduced UACR in patients treated with SGLT2 inhibitors at baseline (25% UACR reduction, 95% CI 10–38) and in those not treated (31% UACR reduction, 95% CI 29–34).^13^ A preclinical rat study of cardio-renal disease demonstrated that combination therapy with empagliflozin and finerenone resulted in a synergistic effect on albuminuria lowering and improved survival compared with either therapy alone.^14^

MRAs increase serum potassium concentration and the risk of hyperkalemia. These effects are more pronounced in patients with CKD and heart failure, who may be most apt to benefit from these therapies. In contrast, SGLT2 inhibitors reduce the risk of hyperkalemia. For example, in patients with type 2 diabetes and CKD participating in the CREDENCE trial, canagliflozin reduced the risk of investigator-reported hyperkalemia-related AEs, initiation of potassium-sparing medications, and serum potassium concentrations ≥6.0 mmol/l.^15^ Moreover, in the DAPA-HF trial, among patients with heart failure and reduced ejection fraction who were receiving MRAs, dapagliflozin reduced the incidence of hyperkalemia.^16^ In the DAPA-CKD trial, numerically fewer patients experienced hyperkalemia irrespective of baseline MRA use. These data provide a compelling rationale to test the long-term efficacy and safety of combination SGLT2 inhibitor—MRA therapy in patients with CKD and heart failure.

This study has some limitations. There were relatively few participants treated with MRAs at baseline, which limits the precision of the effect estimates. In addition, we were unable to assess the effect of initiation of combination therapy because participants were on a stable dose of MRAs before enrolment into the trial. As a result, we were unable to test the hypothesis that dapagliflozin in combination with MRA treatment results in additive or synergistic effects. Nevertheless, the nearly identical effect sizes suggest that there are independent effects of both agents in this population.

In conclusion, in patients with CKD with and without type 2 diabetes, the effects of dapagliflozin in reducing the incidence of the primary composite and composite kidney end points, and of attenuating the decline in eGFR slope, were present in patients treated or not treated with MRAs at baseline. These findings support assessment of combination therapy with MRAs and SGLT2 inhibitors in future clinical trials.

## Disclosure

MP, PV, and NJ have nothing to declare. BVS, AML, and CDS are employees and stockholders of AstraZeneca. GMC has received fees from AstraZeneca for the DAPA-CKD Trial Steering Committee, research grants from the National Institute of Diabetes and Digestive and Kidney Diseases, support for research staff attending meetings from Amgen, participated in data safety monitoring boards for Angion, Bayer, and Recor, is on the board of directors for Satellite Healthcare and on trial steering committees for Akebia, Gilead, Sanifit, and Vertez, and holds stock options with Ardelyx, CloudCath, Durect, DxNow, Miromatrix, Outset, and Unicycive. JJVM has received payments to his employer, Glasgow University, for his work on clinical trials, consulting, and other activities from AstraZeneca, Cytokinetics, KBP Biosciences, Amgen, Bayer, Theracos, Ionis Pharmaceuticals, Dalcor Pharmaceuticals, Novartis, GlaxoSmithKline, Bristol Myers Squibb, Boehringer Ingelheim, Cardurion, and Alnylam, and has received personal lecture fees from Abbott, Alkem Metabolics, Eris Life Sciences, Hickma, Lupin, Sun Pharmaceuticals, Medscape/Heart.org, ProAdWise Communications, Radcliffe Cardiology, Servier, and the Corpus. RC-R is a member of the Executive Committee of the DAPA-CKD study and has received grants/contracts from GlaxoSmithKline and Novo Nordisk, consulting fees from Boehringer Ingelheim and Chinook, and payment/honoraria as a speaker or advisor from Boehringer Ingelheim, Amgen, Janssen, and Novo Nordisk. PR has received honoraria to Steno Diabetes Center Copenhagen for steering group membership and/or lectures and advice from AstraZeneca, Novo Nordisk, Bayer, and Eli Lilly; advisory board participation from Sanofi Aventis and Boehringer Ingelheim; and steering group participation from Gilead. RDT is a member of the Executive Committee of the DAPA-CKD study, has received consulting fees from Boehringer Ingelheim, Reata Pharma, and Chinook and payment/honoraria from Medscape and Medical Education Resources, participated in advisory boards for Bayer and Vifor and on data monitoring committees for Akebia and Otsuka. DCW has received consultancy fees from AstraZeneca and personal fees from Bayer, Boehringer Ingelheim, Astellas, GlaxoSmithKline, Janssen, Napp, Mundipharma, Reata, Vifor Fresenius, and Tricida. HJLH has received funding/honoraria and consulting fees to his institution for Steering Committee membership and/or advisory board participation from AstraZeneca (DAPA-CKD study), AbbVie, Travere Pharmaceuticals, Janssen, Gilead, Bayer, Chinook, Merck, and CSL Pharma, consulting fees from Boehringer Ingelheim and Novo Nordisk, honoraria for lectures from AstraZeneca, and participated in advisory boards for Mitsubishi Tanabe and Mundipharma.

## Supplementary Material

Supplementary File (Word)

## References

[1] Muskiet M.H.A., Wheeler D.C., Heerspink H.J.L. (2019). New pharmacological strategies for protecting kidney function in type 2 diabetes. Lancet Diabetes Endocrinol.

[2] Mehdi U.F., Adams-Huet B., Raskin P. (2009). Addition of angiotensin receptor blockade or mineralocorticoid antagonism to maximal angiotensin-converting enzyme inhibition in diabetic nephropathy. J Am Soc Nephrol.

[3] Bakris G.L., Agarwal R., Anker S.D. (2020). Effect of finerenone on chronic kidney disease outcomes in Type 2 diabetes. N Engl J Med.

[4] Pitt B., Filippatos G., Agarwal R. (2021). Cardiovascular events with finerenone in kidney disease and type 2 diabetes. N Engl J Med.

[5] Perkovic V., Jardine M.J., Neal B. (2019). Canagliflozin and renal outcomes in type 2 diabetes and nephropathy. N Engl J Med.

[6] Heerspink H.J.L., Stefansson B.V., Correa-Rotter R. (2020). Dapagliflozin in patients with chronic kidney disease. N Engl J Med.

[7] Wheeler D.C., Stefansson B.V., Jongs N. (2021). Effects of dapagliflozin on major adverse kidney and cardiovascular events in patients with diabetic and non-diabetic chronic kidney disease: a prespecified analysis from the DAPA-CKD trial. Lancet Diabetes Endocrinol.

[8] Heerspink H.J.L., Stefansson B.V., Chertow G.M. (2020). Rationale and protocol of the Dapagliflozin and Prevention of Adverse outcomes in Chronic Kidney Disease (DAPA-CKD) randomized controlled trial. Nephrol Dial Transplant.

[9] Sen T., Heerspink H.J.L. (2021). A kidney perspective on the mechanism of action of sodium glucose co-transporter 2 inhibitors. Cell Metab.

[10] Laursen J.C., Søndergaard-Heinrich N., de Melo J.M.L. (2021). Acute effects of dapagliflozin on renal oxygenation and perfusion in type 1 diabetes with albuminuria: a randomised, double-blind, placebo-controlled crossover trial. EClinicalmedicine.

[11] Bamberg K., Johansson U., Edman K. (2018). Preclinical pharmacology of AZD9977: a novel mineralocorticoid receptor modulator separating organ protection from effects on electrolyte excretion. PLoS One.

[12] Droebner K., Pavkovic M., Grundmann M. (2021). Direct blood pressure-independent anti-fibrotic effects by the selective nonsteroidal mineralocorticoid receptor antagonist finerenone in progressive models of kidney fibrosis. Am J Nephrol.

[13] Rossing P., Filippatos G., Agarwal R. Finerenone in predominantly advanced CKD and Type 2 diabetes with or without sodium-glucose Cotransporter-2 inhibitor therapy. *Kidney Int Rep*. 10.1016/j.ekir.2021.10.008.

[14] Kolkhof P., Hartmann E., Freyberger A. (2021). Effects of finerenone combined with empagliflozin in a model of hypertension-induced end-organ damage. Am J Nephrol.

[15] Neuen B.L., Oshima M., Perkovic V. (2021). Effects of canagliflozin on serum potassium in people with diabetes and chronic kidney disease: the CREDENCE trial. Eur Heart J.

[16] Shen L., Kristensen S.L., Bengtsson O. (2021). Dapagliflozin in HFrEF patients treated with mineralocorticoid receptor antagonists: an analysis of DAPA-HF. JACC Heart Fail.

