# Supplementary material for: The Kidney Protective Effects of the Sodium–Glucose Cotransporter-2 Inhibitor, Dapagliflozin, Are Present in Patients With CKD Treated With Mineralocorticoid Receptor Antagonists
Source: Kidney Int Rep. 2021 Dec 14;7(3):436–43. doi: 10.1016/j.ekir.2021.12.013 (PMC8897688; doi:10.1016/j.ekir.2021.12.013)
Supplement: Supplementary File (Word) [file mmc1.docx]

Supplementary appendices: **The Kidney Protective Effects of the Sodium–Glucose Cotransporter-2 Inhibitor, Dapagliflozin, are Present in Patients with CKD Treated with Mineralocorticoid Receptor Antagonists**

**Appendix 1: Member of various DAPA-CKD committees**

1. DAPA-CKD Executive Committee: Hiddo J.L. Heerspink, David C. Wheeler, Glenn Chertow, Ricardo Correa-Rotter, Tom Greene, Fan Fan Hou, John McMurray, Peter Rossing, Robert Toto, Bergur Stefansson, and Anna Maria Langkilde
2. DAPA-CKD Independent Data Monitoring Committee: Marc A. Pfeffer, Stuart Pocock, Karl Swedberg, Jean L. Rouleau, Nishi Chaturvedi, Peter Ivanovich, Andrew S. Levey, and Heidi Christ-Schmidt
3. DAPA-CKD Event Adjudication Committee: Claes Held, Christina Christersson and Johannes Mann

**Appendix 2: DAPA-CKD Investigator List**

Argentina:

L. E. Maffei, Centro Medico Dra Laura Maffei, Buenos Aires; P. Raffaele, Fundacion Favaloro, Ciudad Autonoma Buenos Aires; S. E. Solis, Centro Diabetologico, Cordoba; C. A. Arias, CEMEDIC, Ciudad Autonoma Buenos Aires; D. Aizenberg, Centro Medico Viamonte, CABA; C. Luquez, Centro Medico Luquez, Cordoba; C. Zaidman, CIPREC, CABA; N. Cluigt, Instituto de Investigaciones Clinicas Mar del Plata, Mar del Plata; M. Mayer, Centro de Salud e Investigaciones Medicas, Santa Rosa; A. Alvarisqueta, Centro de Investigaciones Medicas, Mar del Plata; A. Wassermann, FEPREVA, CABA; R. Maldonado, Clinica Privada Velez Sarsfield, Cordoba; J. Bittar, Renal SRL Centro Privado De Nefrologia, San Luis; M. Maurich, Instituto de Cardiologia de Corrientes 'J.F.Cabral', Corrientes; L. E. Gaite, Clinica de Nefrologia, Santa Fe; N. Garcia, Blossom DMO, Cordoba; L. Sivak, GEMA Consultorios Medicos, Buenos Aires; P. O. Ramallo, Centro Modelo de Cardiologia, San Miguel de Tucuman; J. C. Santos, Investigaciones Clinicas Tucuman, San Miguel de Tucuman; R. Garcia Duran, Instituto de Investigaciones Clinicas San Nicolas, San Nicolas; J. A. Oddino, Insituto Medico de la Fundacion Estudios Clinicos, Rosario; A. Maranon, Consultorios Medicos Belloni, Nueve de julio

Brazil:

L. N. Maia, Hospital de Base Sao Jose do Rio Preto, Sao Jose do Rio Preto; D. D Avila, Hospital Sao Lucas da PUCRS, Porto Alegre; E. J. G. Barros, Hospital de Clinicas de Porto Alegre, Porto Alegre; M. H. Vidotti, Loema - Instituto de Pesquisa Clinica, Campinas; D. Panarotto, IPCEM - Centro de Ciencias da Saude -Universidade de Caxias, Caxias do Sul; I. D. L. Noronha, Hospital das Clinicas da FMUSP, Sao Paulo; L. A. A. Turatti, CPQuali Pesquisa Clinica Ltda, Sao Paulo; L. Deboni, Fundacao Pro-Rim, Joinville; M. E. Canziani, Hospital do Rim e Hipertensao - UNIFESP, Sao Paulo; M. C. Riella, Instituto Scribner, Curitiba; M. R. Bacci, Praxis Pesquisa Medica, Santo Andre; R. P. Paschoalin, Centro de Estudos Clinica Senhor do Bonfim, Salvador; R. J. Franco, Universidade Estadual de Sao Paulo, Botucatu; J. C. Goldani, Irmandade Santa Casa de Misericordia de Porto Alegre, Porto Alegre

Canada:

E. St-Amour, Q and T Research Outaouais Incorporated, Gatineau; A. W. Steele, Lakeridge Health Oshawa, Oshawa; R. Goldenberg, LMC Clinical Research Inc. (Thornhill), Concord; S. Pandeya, Pandeya Kidney Centre, Oakville; H. Bajaj, LMC Clinical Research Inc, Brampton; D. Cherney, The Toronto Hospital, Toronto; S. M. Kaiser, Nova Scotia Health Authority, Halifax; J. R. Conway, Diabetes Clinic, Smiths Falls; S. S. Chow, Stephen S. Chow Medicine Professional Corporation, Toronto; G. Bailey, Red Deer Medical Centre, Red Deer; J. Lafrance, CIUSSS-estmtl Hopital Maisonneuve-Rosemont, Montreal; J. Winterstein, C-health, Edmonton; S. Cournoyer, CISSSMC - Hospital Charles Le Moyne, Greenfield Park; D. Gaudet, ECOGENE-21, Chicoutimi; F. Madore, Hopital du Sacre-Coeur de Montreal, Montreal; R. L. Houlden, Kingston Health Sciences Centre, Kingston; A. Dowell, Dynamik Research Inc, Pointe-Claire; M. Langlois, CHUS - Hospital Fleurimont, Sherbrooke; N. Muirhead, London Health Sciences Centre, London; H. Khandwala, LMC Clinical Research Inc (Etobicoke), Etobicoke; A. Levin, St Pauls Hospital, Vancouver

China:

F. Hou, Nanfang Hospital of Nanfang medical university, Guangzhou; Y. Xue, Nanfang Hospital of Nanfang medical university, Guangzhou; L. Zuo, Peking university people‘s hospital, Beijing; C. Hao, Huashan Hospital Affiliated to Fudan University, Shanghai; Z. Ni, Renji Hospital Affiliated to Shanghai Jiaotongl Univ., Shanghai; C. Xing, NANJING, Nan Jin; N. Chen, Ruijin hospital Shanghai Jiaotong University of medicine, Shanghai; Y. Dong, The First Affiliated Hospital of Sun Yat-sen University, Guangzhou; R. Zhou, Yangpu Hospital, Shanghai; X. Xiao, Xiangya Hospital Central-south University, Changsha; Y. Zou, Sichuan Academy of Medical Sciences&Sichuan Provincial Peopl, Chengdu; C. Wang, The First Affiliated Hospital of Baotou Medical College, Baotou; B. Liu, Zhongda Hospital Southeast University, Nanjing; Q. Chen, 1st Affiliated Hospital of Nanchang University, Nanchang; M. Lin, PuAi Hospital of Wuhan City, Wuhan; Q. Luo, HwaMei Hospital,University Of Chinese Academy Of Sciences, Ningbo; D. Zhang, Peking University International Hospital, Beijing; J. Wang, Lanzhou University Second Hospital, Lanzhou; M. Chen, General Hospital of Ningxia Medical University, Yinchuan; X. Wang, The First People's Hospital of YueYang, Yueyang; A. Zhong, Jiangxi Provincial People's Hospital, Nanchang; J. Dong, PuAi Hospital of Wuhan City, Wuhan; C. Zhu, Affiliated Hospital of Guizhou Medical University, Guiyang; T. Yan, Tianjin Medical University General Hospital, Tianjin; P. Luo, 2nd hospital of Jilin University, Changchun; Y. Ren, West China Hospital, Chengdu; P. Pai, The University of Hong Kong - Shenzhen Hospital, Shenzhen; D. Li, Shengjing Hospital of China Medical University, Shengyang; R. Zhang, Jilin Province people's hospital, Changchun; J. Zhang, 1st Affiliated Hospital of Beijing University, Beijing; M. Xu, Sun Yat-Sen Memorial Hospital, Guangzhou; Y. Zhuang, 900 Hospital of the Joint Logistics Team, Fuzhou; Y. Kong, Foshan 1st people hospital, Foshan; X. Yao, Jilin Central Hospital, Jilin; X. Peng, The People’s Hospital of Guangxi Zhuang Autonomous Region, Nanning

Germany:

H. Haller, Medizinische Hochschule Hannover, Hannover; G. Klausmann, Studienzentrum Aschaffenburg, Aschaffenburg; D. Tschope, Herz- und Diabeteszentrum NRW, Bad Oeynhausen; T. Kruger, Gemeinschaftspraxis Karlstrasse, Dusseldorf; P. Gross, Studienzentrum Metabolisch-Vaskulare Medizin, Dresden; C. Hugo, Universitatsklinikum Carl Gustav Carus der TU Dresden, Dresden; N. Obermuller, Klinikum der Johann Wolfgang Goethe Universitaet, Frankfurt am Main; L. Rose, Zentrum fur Diabetes und, Munster; P. Mertens, Otto-von-Guericke Universitat Magdeburg, Magdeburg; H. Zeller-Stefan, InnoDiab Forschung GmbH, Essen; A. Fritsche, Klinikum der Eberhard-Karls-Universitat Tubingen, Tubingen; L. Renders, Klinikum rechts der Isar der Technischen Universitat, Munchen; J. Muller, Ambulanzentrum Dr. Mueller Dr. Appelt, Schweinfurt; K. Budde, Charite - Universitatsklinikum Berlin, Berlin; B. Schroppel, Universitaetsklinikum Ulm, Ulm

Denmark:

F. I. Persson, Steno Diabetes Center Copenhagen, Gentofte; T. K. Hansen, Aarhus Universitetshospital, Aarhus; R. Borg, Sjaellands Universitetshospital, Roskilde; U. Pedersen Bjergaard, Nordsjaellands Hospital Hillerod, Hillerod; D. Hansen, Herlev Hospital, Herlev; M. Hornum, Rigshospitalet, Copenhagen

Spain:

V. Escudero Quesada, Hospital Universitario Dr. Peset, Valencia; C. Barrios Barrea, Barcelona,H. del Mar,Nefrologia, Barcelona; E. Espinel Garauz, Barcelona,H. Vall d'Hebron,Nefrologia, Barcelona; J. M. Cruzado Garrit, H.Llobregat, L'Hospitalet de Llobregat; C. Morales Portillo, Hospital Universitario Virgen Macarena, Sevilla; J. L. Gorriz Teruel, Hospital Clinico Universitario de Valencia, Valencia; S. Cigarran Guldris, Hospital Publico de Marina, Burela (Lugo); M. Praga Terente, Madrid, H. 12 de Octubre,Nefrologia, Madrid; N. R. Robles Perez-Monteoliva, Badajoz, H. Infanta Cristina, Nefrologia, Badajoz; F. J. Tinahones Madueno, Hospital Clinico Virgen de la Victoria, Malaga; A. Soto Gonzalez, Complejo Hospitalario Univeritario A Coruna, La Coruna; C. Diaz Rodriguez, Hospital Clinico Universitario Santiago de Compostela, Santiago de Compostela

United Kingdom:

P. Mark, Queen Elizabeth University Hospital, Glasgow; J. Barratt, Leicester General Hospital, Leicester; S. Bhandari, Hull and East Yorkshire Hospitals NHS Trust, Hull; D. Fraser, UNIVERSITY HOSPITAL OF WALES, CARDIFF; P. Kalra, Salford Royal NHS Foundation Trust, Salford; S. P. Kon, King's College Hospital, London; K. Mccafferty, Barts Health NHS Trust - St Bartholomew's Hospital, London; A. Mikhail, Morriston Hospital, Swansea; S. P. Kon, Princess Royal University Hospital (GI), Kent

Hungary:

I. Wittmann, Pecsi Tudomanyegyetem, Pecs; P. Voros, Del-pesti Centrumkorhaz - Orszagos Hemat. es Infekt. Intezet, Budapest; M. Dudas, Bekes Megyei Kozponti Korhaz, Gyula; G. A. Tabak, Semmelweis Egyetem I Belgyogyaszati Klinika, Budapest; R. Kirschner, Flor Ferenc Korhaz, Kistarcsa; A. Letoha, SZTE AOK I. Belklinika, Szeged; I. Balku, Szabolcs-Szatmar-Bereg Megyei KHk es Egyetemi Oktatokorhaz, Nyiregyhaza; Z. Hermanyi, Bajcsy-Zsilinszky Korhaz- es Rendelointezet, Budapest; G. Zakar, Velencei-tavi Jarobeteg Szakellato Kozhasznu Nonprofit Kft, Velence; I. Mezei, Markhot Ferenc Oktatokorhaz Csecsemo- es Gyermekgyogyaszati, Eger; G. G. Nagy, Borsod-Abauj-Zemplen Megyei Korhaz es Egyetemi Oktatokorhaz, Miskolc; J. Lippai, Toth Ilona Egeszsegugyi Szolgalat, Budapest; A. Nemeth, Kanizsai Dorottya Korhaz, Nagykanizsa

India:

D. Khullar, Max Super Speciality Hospital, New Delhi; P. K. Gowdaiah, Bangalore Medical College & Research Institute, Bangalore; E. Fernando Mervin, Govt. Stanley medical college & Hospital, Chennai; V. A. Rao, St. Theresa's Hospital, Hyderabad; D. Dewan, Ajanta Hospital and IVF Center, Lucknow; K. Goplani, B. J. Medical College & Civil Hospital, Ahmedabad; V. S. K. Maddi, Sunrise Hospitals, Vijayawada; M. S. Vyawahare, Government Medical College, Nagpur; R. K. Pulichikkat, Sree Narayana Institute of Medical Sciences, Ernakulam; R. Pandey, Institute of Post Graduate Medical Education & Research, Kolkata; S. K. Sonkar, King George's Medical University, Lucknow; V. K. Gupta, Ganesh Shankar Vidyarthi Memorial Medical College, Kanpur; S. Agarwal, Ruby Hall Clinic, Pune; A. J. Asirvatham, Arthur Asirvatham Hospital, Madurai; A. Ignatius, Noble Hospital, Maharashtra; S. Chaubey, Meditrina Institute of Medical Sciences, Nagpur; S. Melemadathil, Calicut Medical College, Calicut; H. Alva, Vinaya Hospital and Research Centre, Mangalore; Y. Kadam, CIMET's Inamdar Multispeciality Hospital, Pune

Japan:

H. Shimizu, Kojunkai Daido Clinic, Nagoya-shi; A. Sueyoshi, Uji-Tokushukai Medical Center, Uji-shi; H. Takeoka, Hyogo Prefectural Amagasaki General Medical Center, Amagasaki-shi; Y. Abe, Abe Diabetes Clinic, Oita-shi; T. Imai, JA Toride Medical Center, Toride-shi; Y. Onishi, The institute for adult diseases Asahi Life Foundation, Chuo-ku; Y. Fujita, Chubu Rosai Hospital, Nagoya-shi; Y. Tokita, Fujisawa City Hospital, Fujisawa-shi; M. Oura, Yaizu City Hospital; Y. Makita, Koshigaya Municipal Hospital, Koshigaya-shi; A. Idogaki, Medical corporation Tokushukai Nozaki Tokushukai Hospital, Daito-shi; R. Koyama, Tsuchiura Kyodo General Hospital, Tsuchiura-shi; H. Kikuchi, National Hospital Organization Beppu Medical Center, Beppu-shi; N. Kashihara, Kawasaki Medical School Hospital, Kurashiki; T. Hayashi, Osaka General Medical Center, Osaka-shi; Y. Ando, National Hospital Organization Osaka Minami Medical Center, Kawachinagano-shi; T. Tanaka, National Hospital Organization Mie Chuo Medical Center, Tsu-shi; M. Shimizu, National Hospital Organization Kobe Medical Center, Kobe-shi; S. Hidaka, Shonan Kamakura General Hospital, Kamakura-shi; T. Gohda, Juntendo University Hospital, Bunkyo-ku; K. Tamura, Yokohama City University Hospital, Yokohama-shi; M. Abe, Nihon University Itabashi Hospital, Itabashi-ku; Y. Kamijo, Shinshu University Hospital, Matsumoto-shi; T. Imasawa, National Hospital Organization Chiba East Hospital, Chiba-shi; Y. Takahashi, National Hospital Organization Shinshu Ueda Medical Center, Ueda-shi; M. Nakayama, National Hospital Organization Kyushu Medical Center, Fukuoka-shi; M. Tomita, National Hospital Organization Kumamoto Medical Center, Kumamoto-shi; F. Hirano, National Hospital Organization Asahikawa Medical Center, Asahikawa-shi; M. Nakayama, Nakayama Clinic, Nagoya-shi; Y. Fukushima, Fukuwa Clinic, Chuo-ku; A. Kiyosue, Tokyo Eki Center-Building Clinic, Chuo-ku; S. Kurioka, Medical Corporation Kyoujinkai Clinic Komatsu, Neyagawa-shi; E. Imai, Nakayamadera Imai Clini, Takarazuka-shi; K. Kitagawa, National Hospital Organization Kanazawa Medical Center, Kanazawa-shi; M. Waki, Shizuoka City Shizuoka Hospital, Shizuoka-shi; J. Wada, Okayama University Hospital, Okayama-shi; K. Uehara, Naha City Hospital, Naha-shi; H. Iwatani, National Hospital Organization Osaka National Hospital, Osaka-shi; K. Ota, National Hospital Organization Okayama Medical Center, Okayama-shi; S. Shibazaki, National Hospital Organization Hokkaido Medical Center, Sapporo-shi; K. Tamura, Shinonoi General Hospital, Nagano-shi; K. Katayama, Mie University Hospital, Tsu-shi; I. Narita, Niigata University Medical and Dental Hospital, Niigata-shi; M. Iinuma, National Hospital Organization Mito Medical Center, Higashiibaraki-gun; S. Matsueda, National Hospital Organization Fukuoka-Higashi Medical Cente, Koga-shi; S. Sasaki, Iizuka Hospital, Iizuka-shi; A. Yokochi, JOHAS, Kanto Rosai Hospital, Kawasaki-shi; T. Tsukamoto, The TazukeKofukai Medical Research Institute Kitano Hospital, Osaka-shi; T. Yoshimura, Saga-Ken Medical Centre Koseikan, Saga-shi

Korea:

S. Kang, Yonsei University Severance Hospital, Seoul; S. Lee, Ewha Womans University Mokdong Hospital, Seoul; C. S. Lim, SMG - SNU Boramae Medical Center, Seoul; H. Chin, Seoul National University Bundang Hospital, Seongnam-si; K. W. Joo, Seoul National University Hospital, Seoul; S. Y. Han, Inje University Ilsan Paik Hospital, Goyang-si; T. I. Chang, National Health Insurance Service Ilsan Hospital, Goyang-si; S. Park, Kyungpook National University Hospital, Deagu; H. Park, Gangnam Severance Hospital, Seoul; C. W. Park, The Catholic University of Korea, Seoul; B. G. Han, Wonju Severance Christian Hospital, Wonju-si; D. R. Cha, Korea University Ansan Hospital, Ansan-si; S. A. Yoon, Uijeongbu St. Mary's Hospital, Uijeongbu-si; W. Kim, Chonbuk National University Hospital, Jeonju-si; S. W. Kim, Chonnam National University Hospital, Gwangju; D. Ryu, Ewha Womans University Seoul Hospital, Seoul

Mexico:

R. Correa Rotter, Inst Nac de Ciencias Medicas y Nutricion Salvador Zubiran, D.F; S. S. Irizar Santana, C. para el desarrollo de la Med y la asistencia Medica Espec, Culiacan; G. Hernandez Llamas, Estudios Clinicos de Vanguardia, Mazatlan; R. Valdez Ortiz, Hospital General de Mexico Dr. Eduardo Liceaga, Mexico; N. C. Secchi Nicolas, Hospital General de Minatitlan, Minatitlan; G. Gonzalez Galvez, Instituto Jalisciense de Investigacion en Diabetes y Obesida, Guadalajara; J. R. Lazcano Soto, Instituto de Investigaciones Aplicadas a la Neurociencia A.C, Durango; T. Bochicchio Riccardelli, Investigacion Nefrologica S.C., Cuernavaca; E. A. Bayram Llamas, Fundacion Cardiovascular de Aguascalientes, Aguascalientes; D. R. Ramos Ibarra, Centro de Estudios de Alta Especialidad de Sinaloa, Mazatlan; M. G. S. Melo, Clinica Quirurgica de la Concepcion, Saltillo; J. G. Gonzalez Gonzalez, Hospital Universitario 'Dr. Jose Eleuterio Gonzalez', Monterrey; J. H. Sanchez Mijangos, CAIFRC OMEGA SC, Mexico; M. Madero Robalo, Instituto Nacional de Cardiologia, Mexico; A. Garcia Castillo, CardioLink Clin Trials, Monterey

Peru:

H. A. Manrique, Centro de Expertos en Diabetes, Lima; J. C. Farfan, Centro Medico Monte Carmelo, Arequipa; R. Vargas, Centro de Investigacion Endocrino y Transtornos Metabolicos, Piura; A. Valdivia, Hospital Militar Geriatico, Chorrillos; A. Dextre, ENDOMED, San Miguel; E. Escudero, Hospital Nacional Arzobispo Loayza, Lima; J. R. Calderon Ticona, Serv de Endocrinologia y Metabolismo Clinica Senor de los M, Lima; L. Gonzales, Centro de Investigacion de Enf Metabolicas y Cardiologicas, Ica; J. Villena, Hospital Nacional Cayetano Heredia, Lima; L. Leon, Clinica Anglo Americana, Lima; G. Molina, Clinica San Pablo, Lima; A. Saavedra, Centro de Investigacion Ricardo Palma, Lima; E. Garrido, Centro de Investigacion en Endocrinologia, San Isidro; H. Arbanil, Hospital Dos de Mayo, Lima; S. Vargas Marquez, Hospital Nacional Adolfo Guevara Velasco, Cusco; J. Rodriguez, Hospital Alberto Sabogal Sologuren, Callao

Philippines:

R. Isidto, Healthlink Iloilo, Iloilo City; A. J. Villaflor, M3 Dialysis Center, Iloilo; M. A. Gumba, National Kidney and Transplant Institute, Quezon City; L. Tirador, St Pauls Hospital, Iloilo City; R. S. Comia, Amang Rodriguez Medical Center, Marikina; R. A. Sy, Ospital ng Makati Medical Center, Makati City; M. L. V. V. Guanzon, Riverside Medical Center, Bacolod; G. Aquitania, Davao Doctors Hospital, Davao City; N. C. De Asis, Norzel Medical and Diagnostic Clinic, Cebu; A. A. Silva, De La Salle Health Sciences Institute, Dasmarinas City; C. M. Romero, Cebu Doctors' University Hospital, Cebu City; M. E. Lim, East Avenue Medical Center, Quezon City; R. A. Danguilan, National Kidney and Transplant Institute, Quezon City

Poland:

M. Nowicki, SPZOZ Uniwersytecki Szpital Kliniczny, Lodz; H. Rudzki, NZOZ Przychodnia Specjalistyczna Andrzej Wittek, Ruda Slaska; K. Landa, LANDA, NZOZ DIABMED - Spolka Lekarzy Diabetologow, Krakow; I. Kucharczyk-Bauman, Centrum Uslug Medycznych MaxMed Marek Maciaszek, Poznan; B. Gogola-Migdal, Osrodek Badan Klinicznych PARAGON, Bochnia; M. Golski, Ostrowieckie Centrum Medyczne S.C., Bielsko-Biala; A. Olech-Cudzik, Ostrowieckie Centrum Medyczne S.C. Anna Olech-Cudzik, Ostrowiec Swietokrzyski; T. Stompor, Wojewodzki Szpital Specjalistyczny w Olsztynie, Olsztyn; T. Szczepanik, Centrum Medyczne Pratia Katowice, Katowice; B. Miklaszewicz, CARDIAMED Beata Miklaszewicz i Dariusz Dabrowski S.J., Legnica; R. Sciborski, Zespol Opieki Zdrowotnej w Olawie SP ZOZ, Olawa; M. Kuzniewski, CENTERMED Sp. z o.o., Krakow; K. Ciechanowski, Samodzielny Publiczny Szpital Kliniczny Nr2 PUM w Szczecinie, Szczecin; D. Wronska, Profamilia Altera, Katowice; W. Klatko, Specjalistyczny Szpital Wojewodzki w Ciechanowie, Ciechanow; S. Mazur, NZOZ Centrum Medyczne MEDYK, Rzeszow; G. Popenda, NZOZ DIAB SERWIS S.C. Specjalistyczna Przychodnia Lekarska, Chorzow; M. Myslicki, NZOZ Diaverum w Tczewie, Tczew

Russia:

L. Z. Bolieva, North-Ossetian State Medical Academy, Vladikavkaz; S. Berns, Kemerovo Cardiology Dispansery, Kemerovo; A. Galyavich, Kazan State Medical University, Kazan; T. Abissova, Yaroslavl Regional Clinical Hospital, Yaroslavl; I. Karpova, City Diabetology Centre, St.Petersburg; D. Platonov, Tver Regional Hospital, Tver; N. Koziolova, Perm State Medical Academy, Perm; L. Kvitkova, Regional Clinical Hospital, Kemerovo; R. Nilk, City Hospital #38 n.a. N.A.Semashko, Saint Petersburg; T. Medina, Clinical centre of FMBA city of Perm, Perm; A. Rebrov, Regional Clinical Hospital, Saratov; M. Rossovskaya, Karpovich City Clinical Hospital, Krasnoyarsk; I. Sinitsina, Russian Medical postgraduate academy, Moscow; E. Vishneva, City Clinical Hospital 14, Ekaterinburg; N. Zagidullin, Ufa city clinical hosp. 21, Ufa; T. Novikova, St. Petersburg Pokrovskaya City Hospital, Saint-Petersburg; N. Krasnopeeva, Chelyabinsk Road Hospital of RRW, Chelyabinsk; O. Magnitskaya, Volgograd State Medical University, Volgograd; N. Antropenko, Clinical Hospital 1 named after A. N. Kabanov, Omsk; M. Batiushin, Rostov State Medical University, Rostov-on-Don

Sweden:

H. Furuland, Akademiska sjukhuset, Uppsala; A. Saeed, Sahlgrenska Universitetssjukhuse, Goteborg; K. Dreja, Malmo Universitets sjukhus, Malmo; J. Spaak, Danderyds sjukhus AB, Stockholm; A. Bruchfeld, KS Huddinge Hospital, Stockholm

Ukraine:

M. Kolesnyk, State Institution Institute of Nephrology of AMS of Ukraine, Kyiv; O. Levchenko, Municipal Institution 'Odesa Regional Clinical Hospital', Odesa; N. Pyvovarova, Vinnytsya Reg Clin Hospital M.I.Pyrogov - Nephrology, Vinnytsia; V. Stus, CI Regional Clinical Hospital n.a I.I. Mechnikov, Dnipro; V. Doretskyy, ME Volyn Reg Clin Hosp, Lutsk; N. Korobova, ME Rivne Reg Clin Hosp, Rivne; O. Horoshko, MI of Kyiv Regional Council of Kyiv Rerional Hospital #2, Kyiv; I. Katerenchuk, ME Polt Reg Clin Hosp M. V. Skliphosovski Polt Reg Count, Poltava; Y. M. Mostovoy, Private enterprise, Vinnytsia; M. Orynchak, ME Cent City Clin Hosp of Ivano-Frankivsk City Coun-Therap, Ivano-Frankivsk; O. Legun, Ivano-Frankivsk Regional Clinical Hospital, Ivano-Frankivsk; I. Dudar, SI institute of Nephrology NAMS of Ukraine, Kyiv; O. Bilchenko, ME Kharkiv City Clinic of Urgent and Emergency Care-Therap, Kharkiv region; S. Andreychyn, ME Ternopil Municipal City Hospital #2, Ternopil; A. Levchenko, ME 'Reg clin special Center of Ragiation Protection', Kharkiv region; L. Zub, Chernivtsi Regional Clinical Hospital - Internal Medicine, Chernivitsi; N. Tereshchenko, MI Cherkasy Reg Hosp of Cherkasy Regional Council-Nephrology, Cherkasy; I. Topchii, Kharkiv Malaya Institute of Therapy-Nephrology, Kharkiv region; T. Ostapenko, CI Zaporizhzhya Reg Clinical Hospital-Nephrology, Zaporizhzhia; S. Bezuglova, Private medical centre 'OkClinic', Kyiv; M. Kopytsya, Kharkiv Malaya Institute of Therapy, Kharkiv region; O. Turenko, ME Dnipro Multifield Clinical Hospital #4 -Nephrological dep, Dnipro

USA:

O. P. Alvarado, Texas Medical Group of Texas, Fort Worth; R. Anderson, VA Medical Center - NE, Omaha; N. S. Andrawis, Manassas Clinical Research Center, Manassas; A. Arif, Apex Medical Research of Flint PC, Flint; S. A. Benjamin, Universal Research Group, Tacoma; G. Bueso, Endocrine Associates, Houston; R. S. Busch, Albany Medical College, Albany; K. W. Carr, Kenneth W. Carr, MD, Blue Coast Cardiology, Vista; P. Crawford, Research by Design, LLC, Chicago; N. Daboul, Advanced Medical Research, Maumee; G. M. De La Calle, Premier Research Associates, Miami; B. Delgado, San Marcus Research Clinic Inc, Miami Lakes; J. Earl, PMG Research of Hickory, Hickory; M. A. El-Shahawy, Academic Medical Research Institute, Los Angeles; R. J. Graf, MultiCare Research Institute, Tacoma; G. Greenwood, Brookview Hills Research Associates, Winston-Salem; A. Guevara, Alex Guevara DO, Fort Worth; E. M. Wendland, Essentia Health-West Duluth Clinic, Duluth; R. K. Mayfield, Mountain View Clinical Research, Greer; M. Montero, Eastern Nephrology Associates, New Bern; D. J. Morin, Holston Medical Group, Kingsport; P. Narayan, Clinical Research Institute of Northern Virginia, Burke; V. Numrungroad, Suncoast Clinical Research, New Port Richey; A. C. Reddy, Permian Research Foundation, Odessa; R. Reddy, T R Clinic PA, Fort Worth; M. B. Samson, American Clinical Trials, Hawaiian Gardens; R. Trejo, Helix Biomedics LLC, Boynton Beach; M. B. Butcher, Sterling Research Group, Cincinnati; J. K.Wise, Crescent City Clinical Research Center, Metairie; L. R. Zemel, Creekside Endocrine Associates, Denver; M. Raikhel, Torrance Clinical Research, Lomita; D. Weinstein, Zasa Clinical Research, Boynton Beach; P. Hernandez, Elite Clinical Research, Miami; A. Wynne, Cotton O'Neil Clinical Research Center, Topeka; B. V. Khan, Atlanta Vascular Research Foundation (AVRF), Atlanta; G. A. Sterba, Leon Medical Research, Miami; A. Jamal, North America Research Institute, San Dimas; D. Ross, Kansas Nephrology Research Institute/Research Management Inc, Wichita; S. F. Rovner, Academy of Diabetes, El Paso; A. Tan, West Coast Research, San Ramon; F. Ovalle, University of Alabama, Birmingham; R. J. Patel, Lycoming Internal Medicine Inc, Jersey Shore; J. Talano, SWICFT Institute, Naples; D. R. Patel, Southeastern Clinical Research Institute, Augusta; A. Burgner, Nephrology Clinical Trials Center, Nashville; N. Aslam, Mayo Clinic Cancer Center, Jacksonville; M. Elliott, Metrolina Nephrology Associates, Charlotte; S. Goral, University of Pennsylvania, Philadelphia; A. Jovanovich, University of Colorado, Aurora; J. A. Manley, Mountain Kidney & Hypertension Associates, Asheville; K. Umanath, HENRY FORD HOSPITAL, Detroit; D. Waguespack, UT Houston - Texas Medical Center, Houston; D. Weiner, Tufts Medical Center, Boston; M. Yu, Stanford University School of Medicine, Palo Alto; L. Schneider, Tidewater Physicians Multispecialty Group, Newport News; D. Jalal, University of Iowa Hospital & Clinic, Iowa City

Vietnam:

T.Le, T D, Binh Dan Hospital, Ho Chi Minh City; N.Nguyen, T, Thu Duc District hospital, Ho Chi Minh City; H.Nguyen,T T, Dong Nai General Hospital, Bien Hoa; D.Nguyen, B T, Tam Duc hospital, Ho Chi Minh City; V.Nguyen, D K, Bach Mai Hospital, Hanoi; T.Do, G, Bach Mai Hospital, Hanoi; P.Chu, T T, 115 People's Hospital, Ho Chi Minh City; D.Ta, P, 115 Hospital, Ho Chi Minh City; N.Tran, Q, UMC, Ho Chi Minh City; D.Nguyen, A, Director Board, Gia Dinh People Hospital, Ho Chi Minh City; B.Pham, V, Nguyen Tri Phuong Hospital, Ho Chi Minh City
